# The Use of Endothelial Progenitor Cells for the Regeneration of Musculoskeletal and Neural Tissues

**DOI:** 10.1155/2017/1960804

**Published:** 2017-03-28

**Authors:** Naosuke Kamei, Kivanc Atesok, Mitsuo Ochi

**Affiliations:** ^1^Department of Orthopaedic Surgery, Integrated Health Sciences, Institute of Biomedical & Health Sciences, Hiroshima University, Hiroshima, Japan; ^2^Medical Center for Translational and Clinical Research, Hiroshima University Hospital, Hiroshima, Japan; ^3^Section of Orthopaedic Surgery, Spine Fellowship Program, University of Alabama, Birmingham, AL, USA

## Abstract

Endothelial progenitor cells (EPCs) derived from bone marrow and blood can differentiate into endothelial cells and promote neovascularization. In addition, EPCs are a promising cell source for the repair of various types of vascularized tissues and have been used in animal experiments and clinical trials for tissue repair. In this review, we focused on the kinetics of endogenous EPCs during tissue repair and the application of EPCs or stem cell populations containing EPCs for tissue regeneration in musculoskeletal and neural tissues including the bone, skeletal muscle, ligaments, spinal cord, and peripheral nerves. EPCs can be mobilized from bone marrow and recruited to injured tissue to contribute to neovascularization and tissue repair. In addition, EPCs or stem cell populations containing EPCs promote neovascularization and tissue repair through their differentiation to endothelial cells or tissue-specific cells, the upregulation of growth factors, and the induction and activation of endogenous stem cells. Human peripheral blood CD34(+) cells containing EPCs have been used in clinical trials of bone repair. Thus, EPCs are a promising cell source for the treatment of musculoskeletal and neural tissue injury.

## 1. Introduction

Most types of tissue, except the cornea, lens, and cartilage, have blood vessels that supply nutrition. In addition, the vascular niche has been reported recently to play a crucial role in homeostasis, proliferation, and differentiation of somatic stem cells during development and regeneration of tissues [1–8]. Therefore, neovascularization is required not only to supply nutrition but also to improve the environment for the tissue regeneration. The endothelial progenitor cell (EPC) has been reported as a promising cell source for promoting neovascularization [9, 10]. EPCs can differentiate into endothelial cells and contribute directly to the formation of new blood vessels in tumors or ischemic disease [11–14]. On the other hand, EPCs also enhance angiogenesis through the release of proangiogenic factors including vascular endothelial growth factor (VEGF), angiopoietin-1 (Ang1), hepatocyte growth factor (HGF), platelet-derived growth factor (PDGF), monocyte chemotactic protein- (MCP-) 1, and macrophage inflammatory protein- (MIP-) 1 [15–19]. The transplantation of EPCs has been used to treat ischemic diseases in animal models and clinical trials [20–25]. EPCs can also promote the repair of injured tissue through the acceleration of neovascularization. In the present study, we examine the application of EPCs to the repair of musculoskeletal and neural tissues.

## 2. Mobilization and Recruitment of EPCs during Tissue Repair

Circulating EPCs are characterized by the expression of primitive hematopoietic progenitor markers, CD34 or CD133, and endothelial markers, CD31, Flk-1/kinase insert domain receptor (KDR)/VEGF receptor2 (VEGFR2), vascular endothelial- (VE-) cadherin, and Tie2 [26–28]. The EPC colony-forming unit assay of mononuclear cells was developed to assess the quality and quantity of mobilized EPCs [29].

In animal models of bone fracture, enhanced mobilization of bone marrow-derived circulating EPCs and incorporation of the mobilized EPCs into the fracture site have been demonstrated using cell surface markers for EPC [30, 31]. Stromal-derived factor 1 (SDF-1) and CXC chemokine receptor 4 (CXCR4), which is a receptor for SDF-1, play an important role in the mechanism of EPC recruitment for bone fracture healing [32]. Even in humans, the mobilization of EPCs related to osteogenesis has been reported [33]. During distraction osteogenesis of the femur or tibia, the number of CD34(+) or CD133(+)/VEGFR2(+) cells in the peripheral blood mononuclear cell population and the expression level of EPC-mobilizing cytokines including VEGF and SDF-1 in the blood plasma are increased despite no increase in the expression levels of C-reactive protein (CRP). These findings suggest that EPC mobilization is enhanced during osteogenesis without relation to an inflammatory response. Lnk is an adaptor protein and an essential inhibitor of stem cell factor- (SCF-) cKit signaling and thrombopoietin (TPO) signaling during stem cell self-renewal [34–36]. The bone fracture model of Lnk-deficient mice shows accelerated angiogenesis, fracture healing, and remodeling through the enhancement of mobilization and the recruitment of bone marrow EPCs [37]. In addition, Lnk siRNA transfection enhances the function of EPCs for vascularization and improves fracture healing [38]. Therefore, the inhibition of Lnk may have therapeutic potential to enhance fracture healing. The mobilization and incorporation of EPCs have also been reported in a spinal cord injury model using bone marrow transplantation from Tie2/lacZ transgenic mice into wild-type mice [39, 40]. The number of circulating mononuclear cells and EPC colonies formed by the mononuclear cells peaks at day 3 post spinal cord injury, and EPCs recruited into the injured spinal cord markedly increase at day 7 after injury. Even in human spinal cord injury, the number of CD34(+)/CD133(+)/VEGFR2(+) EPCs in peripheral blood is increased within 7 days post injury [41].

## 3. Application of EPCs for Musculoskeletal Tissue Regeneration

### 3.1. Bone Regeneration

Intravenous administration of granulocyte colony-stimulating factor- (G-CSF-) mobilized human peripheral blood CD34(+) cells enhances neovascularization and improves fracture healing in an immune-deficient rat nonhealing femoral fracture model [42]. The results of that study indicate a direct contribution of transplanted CD34(+) cells to vasculogenesis and osteogenesis. The local administration of G-CSF-mobilized human peripheral blood CD34(+) cells with atelocollagen scaffold to the fracture site also results in enhanced angiogenesis, augmented blood flow recovery, and improved fracture healing in the rat nonhealing femoral fracture model (CD34(+) cells were transplanted immediately after the creation of nonhealing fracture) and nonunion model (CD34(+) cells were transplanted at 8 weeks after the creation of the nonhealing fracture) [43, 44]. The transplantation of CD34(+) cells for bone regeneration has been performed in a clinical trial for patients with femoral and tibial nonunion [45, 46]. The intravenous administration of G-CSF-mobilized human peripheral blood CD34(+) cells was also used in immune-deficient rat osteonecrosis models [47]. In that study, the cells inhibited the progression of osteonecrosis after the cauterization of blood vessels at the femoral neck. The transplantation of bone marrow mononuclear cells containing CD34(+) cells has been used to treat patients with idiopathic necrosis at the femoral head [48, 49]. However, the CD34(+) population is rare in mononuclear cells derived from bone marrow or peripheral blood [50, 51]. Therefore, an expansion method of EPCs from CD34(+) was developed [52]. In this method, CD34(+) or CD133(+) cells are cultured in serum-free medium containing VEGF, stem cell factor (SCF), interleukin (IL)-6, Flt3 ligand, and thrombopoietin (TPO). The expansion culture increases CD34(+) or CD133(+) cells that maintain EPC colony-forming potential. Ex vivo expanded CD34(+) cells also show a potential for enhancing bone regeneration [53]. On the other hand, EPCs cultured from bone marrow cells were also reported to enhance bone regeneration in the rat segmental bone defect model [54, 55].

### 3.2. Skeletal Muscle Regeneration

Muscle tissue regeneration induced by EPC transplantation has been reported frequently in ischemic disease models [56–60]. In skeletal muscle injury models, the local transplantation of G-CSF-mobilized human peripheral blood CD133(+) cells enhances angiogenesis, reduces fibrous scar formation, and improves skeletal muscle repair in an immune-deficient rat skeletal muscle injury model [61]. The direct contribution of transplanted human CD133(+) cells to von Willebrand factor- (vWF-) positive blood vessels, desmin- or MyoD1-positive muscle, and Pax7-positive pericyte was shown. However, these cells are rare compared with those on the regenerative area. The expression of VEGF is increased and that of transforming growth factor- (TGF-) *β* is decreased in regenerative tissue after CD133(+) cell transplantation. Therefore, the paracrine effects of transplanted cells may be a main mechanism of skeletal muscle regeneration following CD133(+) cell transplantation. Magnetic targeting was developed as a new cell delivery system using magnetic forces [62]. CD133(+) cells are isolated by the magnetic-activated cell sorting (MACS). Isolated CD133(+) cells are labeled with magnetic beads bound to CD133 antibodies [63]. These CD133(+) cells can be attracted by magnetic forces. The magnetic targeting of G-CSF-mobilized human peripheral blood CD133(+) cells for the immune-deficient rat skeletal muscle injury model demonstrated an improvement in muscle repair even with a small number of CD133(+) cells used in the transplantation [64]. Adipose tissue-derived regenerative cells (ADRCs) can be isolated quickly from harvested adipose tissue using the Celusion system (Cytori Therapeutics, San Diego, USA). ADRCs are heterogenous and contain a rich EPC cell population (CD31+ CD34+ CD45− CD90+ CD105− CD146+) [65]. The local transplantation of human ADRCs to the immune-deficient rat skeletal muscle injury model results in accelerated revascularization and muscle regeneration [66].

### 3.3. Ligament Regeneration

The periodontal ligament (PDL) is a fibrous connective tissue located between the tooth root and the alveolar bone. Fibroblastic lineage cells in PDL tissue have EPC-like properties including the expression of endothelial cell markers and the ability to facilitate the construction of a vascular system [67–69]. CD34(+) cells derived from ruptured human anterior cruciate ligaments (ACL) of the knee also have EPC-like properties and the potential to enhance angiogenesis and osteogenesis [70]. G-CSF-mobilized human peripheral blood CD34(+) cells with atelocollagen were transplanted locally to an immune-deficient rat knee medial collateral ligament injury model [71]. The transplantation of CD34(+) cells increases the expression of VEGF in the injured ligament and promotes the vascularization and repair of ligament tissue. Additionally, combined transplantation of rabbit ligament stem cells and human umbilical cord blood-derived CD34(+) cells improves ligament repair in rat medial collateral ligament injury models [72].

## 4. Application for Neural Tissue Repair

### 4.1. Spinal Cord Regeneration

Human CD34(+) umbilical cord blood cells were used initially as an EPC population for the treatment of spinal cord injury models [73]. In that study, the effect of intraspinal transplantation of CD34(+) on spinal cord repair was compared with that of human bone marrow stromal (BMS) cell transplantation. CD34(+) cell transplantation achieves greater improvement of functional recovery following spinal cord injury compared with that of BMS cell transplantation. Some of the transplanted CD34(+) cells express glial or neural cell markers including glial fibrillary acidic protein (GFAP) or neuronal nuclear antigen (NeuN). However, another study using human CD34(+) umbilical cord blood cells for spinal cord injury models showed that transplanted CD34(+) cells survive in the host spinal cord for at least 3 weeks after transplantation but disappeared by 5 weeks. Additionally, the transplanted cells are not positive for neural markers [74]. Therefore, the differentiation of transplanted CD34(+) cells into glial or neural cells might not be a main mechanism for spinal cord repair. Intravenous administration of G-CSF-mobilized human peripheral blood CD133(+) cells also improves functional recovery in an immune-deficient rat spinal cord injury model [75]. In that study, transplanted CD133(+) cells contribute directly to neovascularization at the injury site. In addition, the expressions of SDF-1 and CXCR4 increase after the CD133(+) cell treatment. Another study also demonstrated that the expressions of SDF-1 and CXCR4 are upregulated after spinal cord injury, along with corresponding trend of endogenous CD133(+) cells with SDF-1 expression [76]. The recruitment of CXCR4(+) cells, including endogenous neural progenitor cells through the SDF-1-CXCR4 axis, may be a mechanism for spinal cord repair after CD133(+) cell treatment. On the other hand, CD133(+) cells derived from human peripheral blood or umbilical cord blood were administrated to an organotypic coculture system consisting of the spinal cord and cortex from neonatal rats [77, 78]. This coculture system allows assessment of the paracrine effect of the transplanted cells on axon growth in the spinal cord [79–81]. The administration of CD133(+) cells increases the expression levels of VEGF, reduces neural apoptosis, and promotes axon growth from the cortex into the spinal cord [77, 78]. The neuroprotective effect and improvement of the microenvironment for axon growth through the upregulation of VEGF expression may be a critical mechanism of CD133(+) cell administration for spinal cord regeneration. Ex vivo expanded CD133(+) human umbilical cord blood cells also show potential for spinal cord regeneration [82]. The ex vivo expansion culture of CD133(+) cells increases the number of cells by 62.8 ± 14.4-fold. Expanded CD133(+) cells have the potential for EPC colony formation and improvement of neovascularization and spinal cord repair similar to fleshly isolated CD133(+) cells. The transplantation of a 20-fold number of expanded CD133(+) cells promotes further angiogenesis, axon growth, and functional recovery in an immune-deficient rat spinal cord injury model [82]. Intravenous transplantation of EPCs cultured from bone marrow mononuclear cells also enhances functional recovery after spinal cord injury [83]. The transplantation of cultured EPCs promotes the repair of injured spinal cord through the induction of astrogliosis and vascular regulation. Astrogliosis in the acute phase of spinal cord injury has been reported to be important for the repair of the blood-brain barrier and for the restriction of inflammation, which leads to a reduction in secondary degeneration after spinal cord injury [84]. A study using Jagged1 knockout mice showed that transplanted EPCs contribute to astrogliosis, vascular regulation, and spinal cord regeneration through the activation of Jagged1-Notch signaling [83]. The knockdown of Lnk in c-Kit(+), Sca-1(−), and lineage marker(−) (KSL) bone marrow stem cell population upregulates the function of these cells as EPCs. Lnk^−/−^ KSL cells form increased numbers of EPC colonies compared to that of Lnk^+/+^ KSL cells. Additionally, the intravenous administration of Lnk^+/+^ KSL cells to a mouse spinal cord injury model promotes angiogenesis, astrogliosis, axon growth, and functional recovery following injury, with Lnk^−/−^ KSL being significantly more effective in inducing and promoting these regenerative events [18]. In all of the above studies, the direct contribution of transplanted EPCs by differentiation to neural cells is very small. However, EPC transplantation promotes neovascularization and induces endogenous CXCR4(+) cell recruitment or astrogliosis. The activation of endogenous stem cells through neurovascular niche formation may be a main mechanism of spinal cord regeneration through EPC transplantation.

### 4.2. Peripheral Nerve Regeneration

The first report of the application of EPCs for the repair of peripheral nerve used the intramuscular injection of EPCs cultured from human cord blood mononuclear cells into hindlimb in streptozotocin-induced diabetic immune-deficient rats [85]. EPC transplantation improves nerve conduction velocity and enhances neovascularization of the hindlimb. On the other hand, peripheral nerve regeneration promoted by the transplantation of G-CSF-mobilized human peripheral blood CD133(+) cells was demonstrated in a sciatic nerve defect model of the immune-deficient rat [86, 87]. In those studies, CD133(+) cells embedded in atelocollagen gel were transplanted into a silicone tube that was then used to bridge a 15 mm defect in the sciatic nerve. Transplantation of CD133(+) cells promotes neovascularization and regeneration of myelinated nerve fibers in the silicone tube. In addition, compound muscle action potentials from hamstring muscle were observed only in CD133(+) cell-treated rats following the electronic stimulation of sciatic nerves. Furthermore, ex vivo expanded CD133(+) cells also show potential for promoting peripheral nerve regeneration similar to freshly isolated CD133(+) cells.

## 5. Conclusions

**Figure 1:**
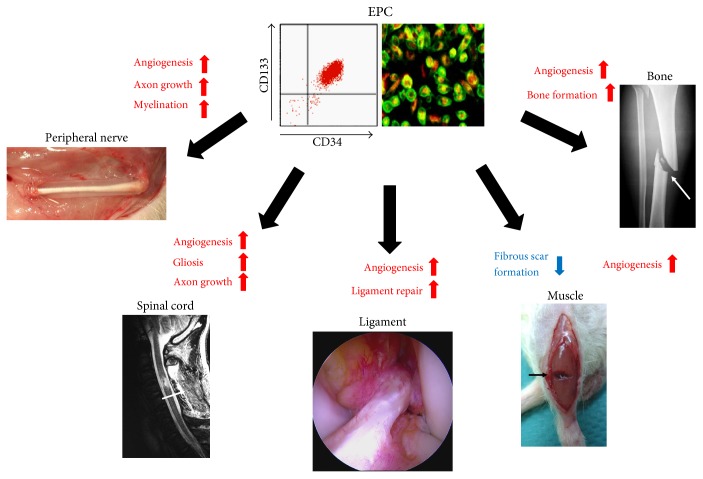
Functions of EPC in the regeneration of musculoskeletal and neural tissues.

During the repair of musculoskeletal and neural tissues including the bone and spinal cord, EPCs are mobilized and recruited to the injured tissue and contribute to neovascularization and tissue repair. The exogenous administration of EPCs or a stem cell population containing EPCs enhances neovascularization and tissue repair. In addition to the direct contribution of EPCs to neovascularization and tissue repair by the differentiation of transplanted EPCs to endothelial cells or tissue-specific cells, the tropic effect derived from transplanted EPCs and the formation of a vascular niche may be important for the tissue repair. From all of these studies, results demonstrate that EPCs are a promising cell source for the treatment of musculoskeletal and neural tissue injury (Figure 1).
